# Meta-Analysis of Phacoemulsification and Laser Peripheral Iridotomy in the Treatment of Primary Angle-Closure Glaucoma

**DOI:** 10.1155/2023/6732424

**Published:** 2023-04-19

**Authors:** Jia Xie, Pengcheng Li, Bo Han

**Affiliations:** Department of Ophthalmology, Union Hospital, Tongji Medical College, Huazhong University of Science and Technology, Wuhan 430022, Hubei Province, China

## Abstract

**Background:**

In this meta-analysis, we aimed to systematically compare the efficacy and safety of phacoemulsification and laser peripheral iridotomy (LPI) in the treatment of primary angle-closure glaucoma (PACG).

**Method:**

We searched PubMed, MEDLINE, EMBASE, the Cochrane Library, the Chinese Journal Full-text Database (CNKI), the Wanfang database, and the China Science and Technology Journal Database for randomized controlled trials (RCTs) of phacoemulsification and LPI in the treatment of PACG published up to September 30, 2022. Postoperative intraocular pressure (IOP), anterior chamber depth (ACD), complications, corneal endothelial cell count, and best-corrected visual acuity (BCVA) were compared. The effective quantity of measurement data was measured by the mean difference (MD) and 95% confidence interval (CI). The effect of counting data was measured by the odds ratio (OR).

**Result:**

A total of 1731 potential studies were identified, and after screening, 8 RCT studies were included. The results of the meta-analysis showed that, compared to the LPI group, the patients in the phacoemulsification group showed lower IOP six and twelve months after operation (MD-3.39, 95% CI −4.15∼−2.63, *P* < 0.00001; −2.29, −3.52∼−1.06, 0.0003). The ACD in the phacoemulsification group was significantly deeper than that in the LPI group (1.59, 1.10∼2.09, 0.00001). Meanwhile, the incidence of complications in the phacoemulsification group was lower than that in the LPI group (OR = 0.46, 0.29∼0.72, 0.0006). There was no statistically significant difference between the phacoemulsification group and the LPI group in corneal endothelial cell count and BCVA at 6 and 12 months after operation (*P*=0.38; 0.11; 0.81).

**Conclusion:**

Compared with LPI, phacoemulsification is safer and more effective in the treatment of PACG, especially in controlling IOP and minimizing postoperative complications.

## 1. Introduction

Glaucoma is one of the leading causes of irreversible blindness worldwide [1]. It was estimated that there were 20.2 million cases of PACG in 2013, and it was projected that this number would rise to 32.04 million in 2040 due to the anticipated increase in the aging population [2]. Although primary open-angle glaucoma (POAG) is more prevalent, PACG is more invasive and on average carries a three-fold higher risk of severe, bilateral visual impairment compared to POAG [3]. The prevalence of glaucoma varied across ethnicity, as PACG is more common in Asians [4]. Moreover, growing amounts of data from recent studies have shown that PACG is 2–4 times more prevalent than previously reported in Europe and the USA [2, 5]. Therefore, it is particularly important to choose the appropriate PACG therapy and treatment.

LPI is commonly performed on PACG patients to relieve pupil blockage. LPI, however, is unable to open the closed anterior chamber angle in up to 58% of PACG patients [6, 7].

Cataract phacoemulsification has improved in recent years due to the development of phacoemulsification and a more reliable fluid flow system [8]. The indications for cataract surgery have been expanded to include the treatment of eye diseases other than cataracts due to improvements in surgical safety. Currently, a growing number of studies indicate that cataract extraction can play a significant role in decreasing IOP and minimizing the application of glaucoma medicines [9–21].

## 2. Methods

### 2.1. Literature Search

Two researchers independently searched the electronic databases such as PubMed, Medline, EMBASE, Cochrane Library, CNKI, Wanfang database, and China Science and Technology Journal Database until September 30^th^ 2022 for studies of RCTs of phacoemulsification and LPI in the treatment of PACG with the following terms: randomized controlled trial, controlled clinical trial, randomized, placebo, randomly, trial, groups, iridotomy, peripheral iridotomy, laser peripheral iridotomy, primary angle closure, angle-glosure glaucoma, angle-closure glaucomas, glaucomas angle closure, glaucoma uncompensated, glaucomas uncompensated, uncompensated glaucoma, ucompensated glaucomas, glaucoma closed-angle, closed-angle glaucoma, closed-angle glaucomas, glaucoma closed angle, glaucomas closed-angle, glaucoma uncompensative, glaucomas uncompensative, uncompensative glaucomas, uncompensative glaucoma, glaucoma angle closure, angle closure glaucoma, angle closure glaucomas, glaucomas angle closure, glaucoma narrow-angle, glaucoma narrow angle, glaucomas narrow-angle, narrow-angle glaucoma, narrow-angle glaucomas, cataract extractions, extraction cataract, extractions cataract, phakectomy, phakectomies, enzymatic zonulolysis, enzymatic zonulolyses, zonulolyses enzymatic, zonulolysis enzymatic, capsulorhexis, and phacoemulsification.

### 2.2. Inclusion and Exclusion Criteria

Studies were included if they met the following criteria: (1) the RCT study of patients with PACG; (2) interventions only included phacoemulsification and LPI; and (3) studies published in Chinese or English.

Studies were excluded if they met the following criteria: (1) they were not RCT studies; (2) studies contained other interventions in addition to phacoemulsification and LPI; (3) studies published in languages other than Chinese or English; and (4) studies with incomplete resources, insufficient data, or lack of author information.

### 2.3. Data Extraction

The relevant data from the articles were extracted by two researchers independently according to the designed data extraction table. The specific information includes the following: (1) general characteristics of the study: including author, publication time, and publication journal; (2) the general characteristics of the subjects included in the study; (3) intervention measures, outcome indicators, etc; and (4) statistical methods of research. Inconsistencies and differences of opinion in the process of extracting data shall be solved through joint discussion with the third researcher.

### 2.4. Assessment of Study Quality

The two researchers independently evaluated the quality of the selected studies according to the evaluation criteria. In case of disagreement, it shall be solved through discussion or by inviting a third researcher. The quality of the RCT study was evaluated by the Cochrane bias risk tool according to the following seven domains: (1) random sequence generation (selection bias); (2) allocation concealment (selection bias); (3) blinding of participants and personnel (implementation bias); (4) blinding of outcome assessment (measurement bias); (5) incomplete outcome data (follow-up bias); (6) selective reporting of research results (reporting bias); and (7) other bias. Each domain was graded into “low risk of bias,” “high risk of bias,” or “unclear risk of bias.”

### 2.5. Statistical Analysis

All analyses were performed with the RevMan5.4 software. The heterogeneity among studies was evaluated by the *Q* test and *I*^2^ test. For the studies with significant heterogeneity (*P* < 0.1 or *I*^2^ > 50%), the random effect model was used for analysis. When significant heterogeneity existed, sensitivity analysis was used to eliminate studies with large differences or studies with a high risk of bias to test the stability of the combined results. For studies with low heterogeneity (*P* > 0.1 or *I*^2^ < 50%), the fixed effect model was used for analysis. IOP, ACD, corneal endothelial cell counting, and BCVA were measured. The mean difference (MD) and 95% confidence interval (CI) were used as statistical analysis variables. The complications during treatment were binary variables, using the odds ratio (OR) and 95% CI as statistical analysis variables. A *P* value less than 0.05 was considered to be statistically significant.

## 3. Results

### 3.1. Search Results

**Figure 1:**
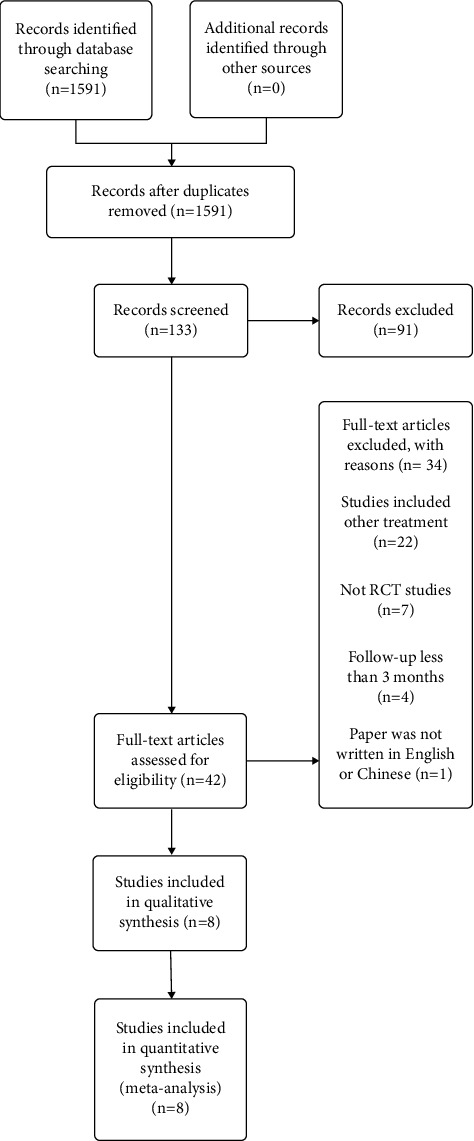
The study flow diagram.

A total number of 1731 potential studies were identified from an electronic-based search, of which 1570 studies remained after eliminating duplicates. Fifty-four studies remained after analyzing the titles and abstracts of the studies and removing any with inconsistent content or incomplete main indicators. Fifty-four full-text articles were identified as potentially relevant for analysis in terms of the inclusion and exclusion criteria. Finally, eight studies [22–29] involving 894 eyes were included in the analysis. There was no significant difference in age, gender composition ratio, and IOP before treatment between the two groups (*P* > 0.05). The article screening process is shown in Figure 1, and the basic information of the included studies is shown in Table 1.

### 3.2. The Characteristics of the Included Studies

**Figure 2:**
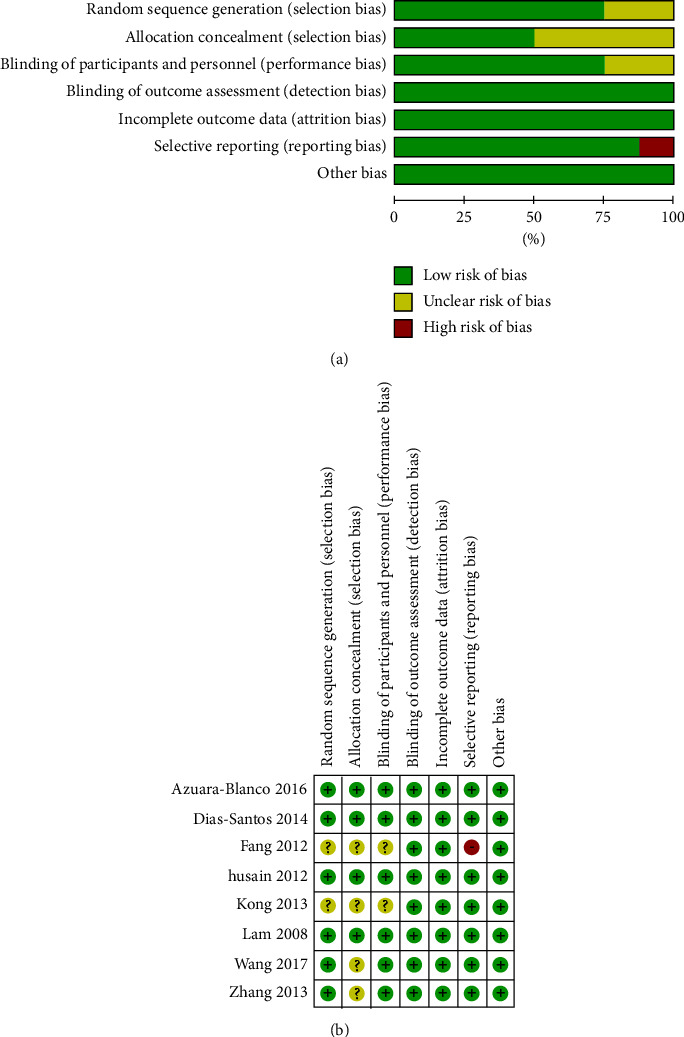
Assessment of the risk of bias in the 8 included RCTs. (a) Judgments about each risk of the bias item presented as percentages across all included studies. (b) Judgments about each risk of the bias item for each included study.

The 8 included studies were all RCTs. The Cochrane Risk of Bias Assessment Tool was used to assess the quality and bias risk of the included RCTs. The results are displayed in Figure 2. Six studies [23, 25–29] mentioned the random sequence generation scheme, indicating a low risk of bias. Two studies [22, 24] were rated as uncertain because the random sequence generation scheme was not specified in the text. Among the selection bias related to allocation concealment, four studies [22–25] did not report it in the paper, so the risk of bias was uncertain. In the implementation bias, two studies [22, 24] could not judge the bias risk, and one study [22] was evaluated as high risk in reporting the source of bias. Among measurement bias, follow-up bias, and other biases, all studies [22–29] were determined as low risk.

### 3.3. Meta-Analysis Results

#### 3.3.1. IOP at Different Times after the Operation

**Figure 3:**
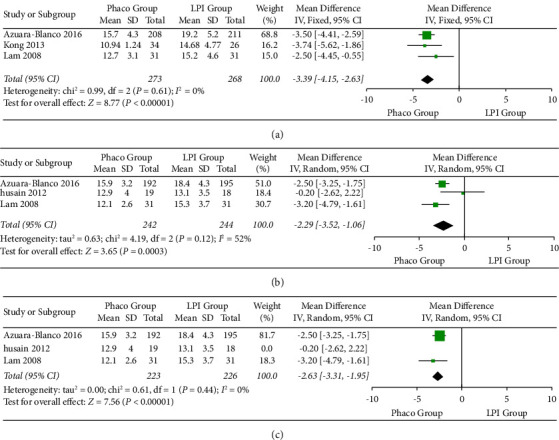
Forest plots comparing the IOP between the Phaco groups and LPI groups at different follow-up times. (a) Six months after the operation. (b) Twelve months after the operation. (c) Twelve months after the operation (excludes the study of Husain et al. [26]).

A total of three studies [24, 27, 29] recorded the IOP values of the two groups six months after treatment. Because no statistical heterogeneity was found among the studies (*P*=0.61 and *I*^2^ = 0%), we used the fixed effect model for analysis. The results showed that the IOP six months after operation in the phacoemulsification group was significantly lower than that in the LPI group, and the difference was statistically significant (MD-3.39, 95% CI −4.15∼−2.63, and *P* < 0.00001; Figure 3(a)).

A total of three studies [26, 27, 29] recorded the IOP values of the two groups of patients twelve months after treatment. There was significant heterogeneity between the studies (*P*=0.12 and *I*^2^ = 52%), so the random effect model was used for analysis. The results showed that the IOP 12 months after operation in the phacoemulsification group was significantly lower than that in the LPI group, and there was statistically significant difference (MD-2.29, 95% CI −3.52∼−1.06, and *P*=0.0003; Figure 3(b)). A sensitivity analysis was conducted for the heterogeneity among studies, and the results showed that the heterogeneity decreased significantly and the combined effect value did not change significantly (*I*^2^ = 0%, MD-2.63, 95% CI −3.31 ∼ −1.95, and *P* < 0.00001; Figure 3(c)) after excluding the study of Husain et al. [26]. It showed that the results of the meta-analysis were stable.

#### 3.3.2. ACD after the Operation

**Figure 4:**
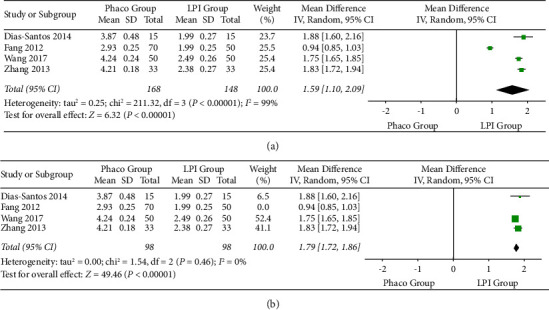
Forest plots comparing the ACD between the Phaco groups and LPI groups after operation. (a) Three months after the operation. (b) Three months after the operation (excludes the study of Min et al. [22]).

A total of four studies [22, 23, 25, 28] recorded the ACD of the two groups three months after the operation. There was significant heterogeneity between the studies (*P* < 0.00001 and *I*^2^ = 99%), so the random effect model was used for analysis. The results showed that the central ACD in the phacoemulsification group was significantly deeper than that in the LPI group three months after the operation, and there was statistically significant difference (MD 1.59, 95% CI 1.10∼2.09, and *P* < 0.00001). A sensitivity analysis was conducted for the heterogeneity among studies, and the results showed that the heterogeneity decreased significantly and the combined effect value did not change significantly (*I*^2^ = 0%, MD 1.79, 95% CI 1.72∼1.86, and *P* < 0.00001) after excluding the study of Min et al. [22]. It showed that the meta-analysis results were stable (Figure 4).

#### 3.3.3. Probability of Complications during Treatment

**Figure 5:**
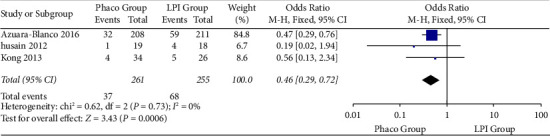
The forest plot comparing the numbers of complications between the Phaco groups and LPI groups.

A total of three studies [24, 26, 29] recorded the complications during postoperative treatment. There was no significant heterogeneity between the studies (*P*=0.73 and *I*^2^ = 0%), so the fixed effect model was used for analysis. The results showed that the incidence of complications in the phacoemulsification group was significantly lower than that in the LPI group, and the difference was statistically significant (OR = 0.46, 95% CI 0.29∼0.72, and *P*=0.0006; Figure 5).

#### 3.3.4. Corneal Endothelial Cell Counting after the Operation

**Figure 6:**
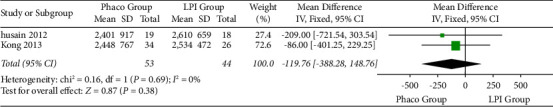
The forest plot comparing corneal endothelial cell count between the Phaco groups and LPI groups at six months after the operation.

A total of two studies [24, 26] recorded corneal endothelial cell counting of the two groups six months after the operation. There was no significant heterogeneity between the studies (*P*=0.69 and *I*^2^ = 0%), so the fixed effect model was used for analysis. The results showed that there was no significant difference in corneal endothelial cell counting between the two groups six months after the operation (*P*=0.38, Figure 6).

#### 3.3.5. BCVA after the Operation

**Figure 7:**
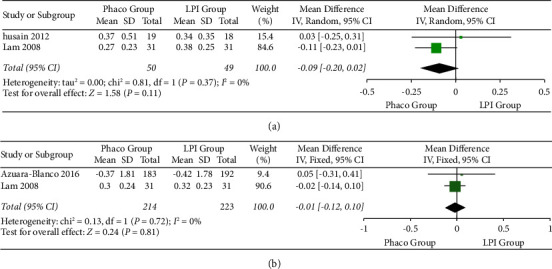
Forest plots comparing the BCVA between the Phaco groups and LPI groups at different follow-up times. (a) Six months after the operation. (b) Twelve months after the operation.

A total of two studies [26, 27] recorded the BCVA of the two groups six months after the operation. There was no significant heterogeneity between the studies (*P*=0.37 and *I*^2^ = 0%), so the fixed effect model was used for analysis. The results showed that there was no significant difference in the BCVA six months after the operation between the two groups (*P*=0.11, Figure 7(a)).

A total of two studies [27, 29] recorded the BCVA of the two groups of patients twelve months after the operation. There was no significant heterogeneity between the studies (*P*=0.72 and *I*^2^ = 0%), so the fixed effect model was used for analysis. The results showed that there was no significant difference in the BCVA twelve months after operation between the two groups (*P*=0.81, Figure 7(b)).

## 4. Discussion

In PACG, the relative pupillary block is a common mechanism that contributes to the closure of the anterior chamber angle, but it is not the only mechanism. According to the World Glaucoma Expert Consensus, [30] the mechanism for angle closure in PACG primarily involves four elements: the iris, ciliary body, lens, and posterior lens. Some patients experience multiple factors contributing to their condition, and lens-related factors play a role in all the mechanisms except the one at the ciliary body level, highlighting the significance of the lens in the pathogenesis of glaucoma.

A multicenter research indicated that clear lens extraction showed greater efficacy and should be considered to be the first-line treatment of PACG [29]. Although phacoemulsification has been used to treat PACG in China 20 years ago, [31] some domestic scholars disagree that clear lens extraction is the first choice for PACG. They believe that LPI can relieve pupil block. As a result, there is no reason to remove the normal lens. It has been suggested that, as an internal eye surgery, the risk of cataract phacoemulsification is higher than that of LPI, there may be more complications, and the visual function of patients may be dramatically worse than before. How should we select a PACG treatment?

Firstly, in most PACG cases, there are some pathological changes even when there is no obvious lens opacity. In these patients, the lens is generally thicker or positioned too far forward, which is a significant pathological mechanism leading to angle closure. Lens extraction can theoretically eliminate the anatomical factors that contribute to this closure. In recent years, several clinical trials have demonstrated that cataract extraction can significantly broaden the anterior chamber angle [32–37]. By comparing ACD between the two groups after treatment, we observed that the ACD in the phacoemulsification group was significantly deeper than that in the LPI group, and the difference was statistically significant, which was consistent with the above research results. These findings confirmed that phacoemulsification could effectively deepen the anterior chamber and inhibit the mechanism of angle closure.

Secondly, our study found that there was no significant difference in corneal endothelial cell count between the two groups six months after treatment (*P*=0.38 > 0.05), indicating that, despite being an internal eye operation, cataract phacoemulsification did not cause additional endothelial cell damage compared to the LPI group. With the advancement of modern cataract phacoemulsification technology and the protection provided by viscoelastic agents, the impact of cataract surgery on the corneal endothelium has been gradually reduced. The results revealed that the occurrence of complications was lower in the phacoemulsification group than that in the LPI group, and the difference was statistically significant (*P*=0.0006 < 0.05). It further proves that phacoemulsification is a safe and effective treatment option for PACG.

At the same time, phacoemulsification has more advantages than LPI in terms of IOP control. At six and twelve months after the operation, the IOP in the phacoemulsification group was lower than that in the LPI group, and the difference was statistically significant.

Finally, this study analyzed the BCVA of the two groups six and twelve months after treatment. The results showed that there was no significant difference in BCVA between the two groups (*P*=0.11 > 0.05), indicating that there were no complications affecting visual acuity in the phacoemulsification. It demonstrated that phacoemulsification did not increase the risk of visual loss while maintaining good IOP control. This further suggests that phacoemulsification is expected to be the first choice for the treatment of angle closure glaucoma.

## 5. Conclusion

Based on the above evidence, cataract phacoemulsification may be a crucial surgical option for the treatment of PACG. Phacoemulsification is more effective than LPI for PACG treatment, particularly in controlling IOP and minimizing complications. However, there are few studies that have examined the effectiveness of the two treatments over the long term. Therefore, additional research is required for long-term follow-up observation to evaluate its safety and validate the advantages already observed so far.

## Figures and Tables

**Table 1:** Characteristics of included studies.

NO	Author	Year	Region	Study design	*No. of eyes*		*Mean age (years)*		Sex: female		Follow-up(months)
					Phaco	LPI	Phaco	LPI	Phaco	LPI	
1	Min et al. [22]	2012	China	RCT	70	50	N/A	N/A	N/A	N/A	3
2	Zhang et al. [23]	2013	China	RCT	33	33	69.04 ± 6.88	72.84 ± 7.05	15	14	3
3	Kong et al. [24]	2013	China	RCT	34	26	67.43 ± 7.43	65.42 ± 6.45	12	13	6
4	Wang [25]	2017	China	RCT	50	50	68.38 ± 3.56	68.54 ± 3.94	19	21	3
5	Husain et al. [26]	2012	Singapore	RCT	19	18	65.9 (42–82)	66.1 (48–85)	16	13	Postoperative day 1, weeks 1, 3, and 6, months 3, 6, 9, and 12, and then every 4 months in year 2
6	Lam et al. [27]	2008	Hong Kong	RCT	31	31	72.3 ± 7.3	69.0 ± 7.8	26	23	day 1; week1; months 1, 3, 6, 9, and 12; and then every 6 months for 4 years
7	Dias-Santos et al. [28]	2014	Portugal	RCT	15	15	69.5 ± 11.34 (52–86)	65.10 ± 9.49 (44–76)	11	12	31.13 ± 4.97
8	Azuara-Blanco et al. [29]	2016	Australia, mainland China, Hong Kong, Malaysia, Singapore, and the UK	RCT	208	211	67·0 (61·0–73·0)	67·0 (61·0–73·0)	122	121	6, 12, 24, and 36 months

## Data Availability

The data used to support the findings of this study are included within the article.
